# Telehealth and virtual supervision practices for health professions education in the Department of Veterans Affairs

**DOI:** 10.1186/s12909-025-06698-7

**Published:** 2025-02-26

**Authors:** Nancy D. Harada, Kimberly Falco, Marjorie Bowman, John M. Byrne

**Affiliations:** 1 U.S. Department of Veterans Affairs, Office of Academic Affiliations, University of California, Los Angeles, David Geffen School of Medicine, 810 Vermont Ave NW, Washington, D.C, 20420 USA; 2 U.S. Department of Veterans Affairs, Office of Academic Affiliations, Nevada State University, School of Nursing, 810 Vermont Ave NW, Washington, D.C, 20420 USA; 3 U.S. Department of Veterans Affairs, Office of Academic Affiliations, Wright State University and the University of Pennsylvania, 810 Vermont Ave NW, Washington, D.C, 20420 USA; 4 U.S. Department of Veterans Affairs, Office of Academic Affiliations, Loma Linda University School of Medicine, 810 Vermont Ave NW, Washington, D.C, 20420 USA

**Keywords:** Telehealth, Virtual supervision, Health professions education

## Abstract

**Background:**

Telehealth and virtual supervision practices in health professions clinical education has grown rapidly, including in the Department of Veterans Affairs (VA) which oversees the largest interprofessional training effort in the United States. Telehealth is the provision of healthcare that is provided remotely through telecommunication technology, and virtual supervision is clinical supervision of health professions trainees (HPTs) that occurs through telecommunication technology. In this study we evaluate participation in telehealth and virtual supervision for physician, nursing, and associated health HPTs, and describe prevalent themes concerning HPT perceptions of telehealth and virtual supervision.

**Methods:**

The survey study cohort included 10,865 HPTs that responded to the VA Trainee Satisfaction Survey in Academic Year 2023 (between July 2022 to June 2023). Descriptive and bivariate analyses were conducted to evaluate participation rates by profession. Responses to the open-ended question within the survey were coded and summarized using rapid qualitative analysis methods.

**Results:**

Participation rates for telehealth and virtual supervision were: Associated health HPTs (47.2% telehealth, 33.8% virtual supervision), physician residents (31.1% telehealth, 21.5% virtual supervision), and nursing HPTs (22.9% telehealth, 21.5% virtual supervision) (*p* < .001). HPTs of all professions expressed positive views on their experiences, with profession-specific differences noted in location, frequency and depth of these practices.

**Conclusions:**

Participation in telehealth and virtual supervision was common and well received by HPTs across multiple professions in VA. HPT responses suggest additional efforts are needed to refine profession-specific instructional methods tailored for defined educational needs and goals.

## Background

Telehealth is provision of healthcare through telecommunication technology. In health professions education (HPE), telehealth is often paired with virtual supervision where health profession trainees (HPTs) receive clinical supervision via communication technology such as telephone, email, or video when the supervisor is not physically present [1–3]. Healthcare trends, including advanced technology and the need for increased healthcare access fueled by the COVID-19 pandemic, have accelerated the adoption of telehealth and virtual supervision, including in the U.S. Department of Veterans Affairs, Veterans Health Administration (VA) [4–9]. In Fiscal Year (FY) 2018, 13% of Veterans received care via telehealth [10], increasing to approximately 40% in FY 2024 [11]. Delivery of care via telehealth occurs across specialties, including 25 medical specialties and 12 rehabilitation specialties including tele-amputation, tele-occupational therapy, and tele-chiropractic care [10, 12]. Additionally, 98% of VA mental health providers have provided at least one video visit within the last year [13]. The rise of telehealth has expanded opportunities for virtual supervision, allowing HPTs to be present where telehealth is delivered.

Managed by the VA Office of Academic Affiliations (OAA), the VA oversees the largest health professions training effort in the United States (U.S.), annually training over 120,000 HPTs from more than 60 professions in 170 VA facilities across the nation [14]. During the COVID-19 pandemic, OAA provided guidance to VA facilities on telehealth and virtual supervision flexibilities necessary for social distancing, enabling remote supervision of HPTs while requiring patient encounters to be discussed with a supervisor, documented in the electronic health record, and co-signed by the supervising practitioner [15, 16].

The VA provides a unique opportunity to evaluate telehealth and virtual supervision across multiple professions in a national, interprofessional, clinical education setting. This paper aims to quantify the participation of HPTs in telehealth and virtual supervision and categorize their perceptions of these practices.

## Methods

### Data source

The data source was the 2023 Trainee Satisfaction Survey (TSS). The TSS is an anonymous, voluntary survey developed by OAA. The survey is administered annually via SurveyMonkey. The survey opens in August of each academic year (AY) and remains open through the following July [17]. HPT’s are requested by their training director each AY to complete the TSS and a reminder is sent out from OAA around March of each AY. The TSS has been used in prior studies to evaluate various aspects of HPT training experiences [6, 18–20].

For this study, all HPTs responding to the survey in AY 2023 (July 1, 2022, to June 30, 2023) are included in the dataset. We estimate a 12% response rate based on the number of responses received and 120,000 total HPTs in AY 2023. HPTs were asked to rate their satisfaction with various aspects of their VA training experience such as the clinical learning and working/physical environments and clinical faculty/preceptors. Respondents were provided with definitions of telehealth and virtual supervision (see below) followed by dichotomous response (yes/no) questions about their participation in telehealth and virtual supervision, and an open-ended question. The open-ended question asked HPTs to “please describe your [virtual supervision] experience”. HPTs were provided with the following definitions on the survey:

- Clinical supervision refers to the interaction between a supervising practitioner and a health professions trainee that is directly related to an encounter, procedure, or episode of care.
- Virtual supervision occurs via phone or video when the supervisor is not physically present with the trainee.
- Telehealth refers to the provision of healthcare remotely by means of telecommunications technology.

The TSS and associated analyses are categorized as an operation’s improvement activity based on Veterans Health Administration (VHA) Handbook 1200.21 [21], where information generated is used for business operations and quality improvement. The overall project is subject to administrative oversight by the federal program office overseeing the TSS, the Office of Academic Affiliations, rather than oversight from a Human Subjects Institutional Review Board.

## Data analysis

### Development of the analytical dataset

The original dataset included 13,995 HPTs throughout the U.S. who responded to the survey during AY 2023. HPTs were excluded from the sample if they stated they did not have a VA training rotation during AY 2023 (*n* = 994),^1^ they did not identify their clinical training profession (*n* = 670), or they did not identify their clinical training facility (*n* = 432). In addition, medical students (*n* = 901) and dental HPTs, e.g. dental students, dental residents, dental assistants and dental hygienists (*n* = 133) were excluded because of their limited telehealth and virtual supervision training experiences.

The final analytical sample consisted of 10,865 HPTs from 133 VA facilities (Table 1). The sample consisted of 43% physician HPTs (both allopathic and osteopathic) (*n* = 4,681), 40% associated health HPTs (*n* = 4,342), and 17% nursing HPTs (*n* = 1,842). Among the nursing HPTs, registered nurses (*n* = 1,246) were the largest group, followed by nurse practitioners (*n* = 371), licensed practical nurses/licensed vocational nurses, nurse assistants (LPN/LVN/NA) (*n* = 125) and others, e.g. certified registered nurse anesthetist or clinical nurse leader (*n* = 100). The top 10 HPT associated health groups were pharmacy (*n* = 850), psychology (*n* = 687), optometry (*n* = 433), physical therapy (*n* = 364), physician assistants (*n* = 353), social work (*n* = 279), occupational therapy (*n* = 205), medical imaging (*n* = 127), dietetics (*n* = 120), and others, e.g. audiology, speech language pathology, and chaplaincy (*n* = 924).

**Table 1:** HPT Clinical Professions in Trainee Satisfaction Survey AY 2022–2023 (*N* = 10,865)

HPT Profession, *n* (% of sample)	*n* (% within profession)
Medical, *n* = 4,681 (43%)	
Physician Resident (MD or DO)	4,681 (100)
Nursing, *n* = 1,842 (17%)	
Registered Nurse	1,246 (67.7)
Nurse Practitioner	371 (20.1)
LPN/LVN/NA	125 (6.8)
Other	100 (5.4)
Associated Health, *n* = 4,342 (40%)	
Pharmacy	850 (19.6)
Psychology	687 (15.8)
Optometry	433 (10.0)
Physical Therapy	364 (8.4)
Physician Assistant	353 (8.1)
Social Work	279 (6.4)
Occupational Therapy	205 (4.7)
Medical Imaging	127 (2.9)
Dietetics	120 (2.8)
Other	924 (21.3)
Total, *N* = 10,865 (100%)	

### Quantitative analyses

Quantitative data were analyzed using SPSS version 29.0.1 [22]. Descriptive statistics were calculated for HPT characteristics including training program type and training year. Bivariate analyses included cross tabulations and chi-square between HPT profession and telehealth or virtual supervision participation.

### Qualitative analyses

Rapid qualitative analysis methods were used to evaluate perceptions of telehealth and virtual supervision across professions [23–25]. Each response was first screened to determine whether the comment addressed (1) telehealth or (2) virtual supervision. Comments that did not address either of these were not included in the qualitative analysis. Doctorally prepared study members (NH, KF) with clinical expertise and comprehensive experience in rapid qualitative analysis worked together to code individual responses to the open-ended survey question where HPTs described their telehealth and virtual supervision experiences. Codes were created inductively from the data to identify recurring patterns and assign descriptive labels as codes (Table 2) [26]. Coders analyzed each response collaboratively and if there were differences on code assignment discussions occurred to come to a consensus. Codes were categorized and grouped to identify main themes, which were then reviewed by the full study team for further discussion and identification of representative quotes.

**Table 2:** Qualitative Analysis – Categories, Codes, and Code Definitions with Examples

Category	Code	Code Definition	Response Word Examples
Telehealth	Neutral to positive	Words aligned with neutral to positive experience	Helpful, enjoyed, effective, good, lack of adjective describing experience
	Negative	Words aligned with negative experience	Useless, time consuming
Virtual supervision	Positive	Words aligned with neutral to positive experience	Convenient, adequate, supportive, lack of an adjective describing experience
	Negative	Words aligned with negative experience	Minimal supervision received, lack of positive and collaborative environment, time consuming
Virtual supervision technology	Technology	HPT named or labeled specific hardware or software technologies	Phone, VA video connect (VVC), Citrix, Laptop, Microsoft Teams
Virtual supervision amount	Amount	The amount of time spent receiving virtual supervision	Frequency Duration Specific rotation e.g. pulmonary clinic rotation
Virtual supervision mode	Audio	Communication with supervisor occurred via audio means	Phone
	Visual	Communication with supervisor occurred via visual means	VVC, Microsoft Teams, Webex
Supervisor	Positive	Supervisor was described in a positive fashion	Kind, supportive, dedicated, attentive
	Negative	Supervisor was described in a negative fashion	Unsupportive, unfocused, disorganized
External factors leading to use of virtual supervision	Factors	Opportunities for training to occur when external events are happening	physical distance between supervisor & HPT (e.g. Covid-19), natural disaster e.g. hurricane

## Results

### Participation in Telehealth and Virtual Supervision by HPT profession

Participation in telehealth and virtual supervision varied significantly by HPT profession (Table 3). Specifically, the highest participation rates were reported by associated health HPTs (47.2% for telehealth, 33.8% for virtual supervision), followed by physician HPTs (31.1% for telehealth, 21.5% for virtual supervision), and nursing HPTs (22.9% for telehealth, 21.5% for virtual supervision). These differences between professions were statistically significant (*p* < 0.001).

**Table 3:** HPT Professions’ Participation in Telehealth and Virtual Supervision. (Trainee Satisfaction Survey, AY 2022-2023)

Profession	*n*	Telehealth Participation^a^ *n* (%)	Virtual Supervision Participation^a^ *n* (%)
Medical	4,681	1,454 (31.1%)	1,005 (21.5%)
Physician Resident (MD or DO)			
Nursing	1,842	421 (22.9%)	263 (14.3%)
Registered Nurse	1,246	130 (10.4%)	68 (5.5%)
Nurse Practitioner	371	266 (71.7%)	175 (47.2%)
LPN/LVN/NA	125	21 (16.8%)	18 (14.4%)
Other	100	3 (3.1%)	1 (1.0%)
Associated Health	4,342	2051 (47.2%)	1466(33.8%)
Pharmacy	850	485 (57.1%)	364 (42.8%)
Psychology	687	645 (93.9%)	590 (85.9%)
Optometry	433	25 (5.8%)	12 (2.8%)
Physical Therapy	364	132 (36.3%)	31 (8.5%)
Physician Assistant	353	75 (21.2%)	34 (9.6%)
Social Work	279	176 (63.1%	130 (46.6%)
Occupational Therapy	205	92 (44.9%)	37 (18.0%)
Medical Imaging	127	3 (2.4%)	2 (1.6%)
Dietetics	120	102 (85.0%)	74 (61.7%)
Other	924	313 (34.2%)	190 (20.8%)
Total	10,865	3926 (36.1%)	2734 (25.2%

^a^Statistically significant differences in telehealth or virtual supervision participation by HPT profession (*p* < .001)

Table 3 provides further detail on participation variations among specific professions. Among nursing HPTs, nurse practitioners had the highest participation rates (71.7% for telehealth, 47.2% for virtual supervision), followed by LPN/LVN/NA (16.8% for telehealth, 14.4% for virtual supervision), and registered nurse HPTs (10.4% for telehealth, 5.5% for virtual supervision). In the associated health professions, psychology HPTs had the highest participation rates in both telehealth (93.9% for telehealth and 85.9% for virtual supervision 85.9%), followed by social work (63.1% for telehealth, 46.6% for virtual supervision), and pharmacy (57.1% for telehealth, 42.8% for virtual supervision).

### Trainee perspectives on telehealth and virtual supervision

There was a total of 1,331 responses to the open-ended question received from HPTs (physician *n* = 338, associated health *n* = 793, nursing *n* = 200). Higher proportions of HPTs expressed neutral to positive views on their experiences with telehealth and virtual supervision in VA facilities. High levels of positivity were expressed in the open-ended comments among nursing HPTs (98.1% for telehealth, 100% for virtual supervision), associated health HPTs (97.1% for telehealth, 97.7% for virtual supervision), and physician HPTs (90.4% for telehealth, 95.8% for virtual supervision). Some associated health and physician HPTs noted, but did not elaborate, that virtual supervision was “as effective as” in-person supervision.

#### Representative comments of neutral to positive virtual supervision experiences

“An attending supervised me either on a separate computer or in the same room as me while I conducted telehealth visits. Overall, it was a similar level of supervision and quality of education in comparison to in-person visits. – Physician HPT

“I met with my preceptor weekly via telehealth. I felt it was just as effective, if not more so, than in person. We did not have technical issues and were able to look at the same patient chart at the same time, which was very helpful.” – Nurse Practitioner HPT

“[Virtual supervision was] convenient with minimal concerns that impacted my training experience, although my preference remains face-to-face supervision. – Psychologist HPT

The quality of the virtual supervision encounters was influenced by technology and supervisory relationships. Technological challenges were frequently associated with negative views of virtual supervision including interruptions in internet connectivity, telephone or video connections, and software issues. Common modalities mentioned included Microsoft Teams, VA Video Connect (VVC), and phone. Additionally, the supervisor-supervisee relationship played an integral role in shaping the virtual supervision experience evidenced by the recurring theme of faculty supervisor traits e.g. accessibility, supervision style influencing the virtual supervision experience. HPTs often commented about faculty supervisors’ traits including supportiveness, resourcefulness, effective communication skills, kindness and attentiveness.

#### Representative comments of virtual supervision influenced by technology

“I felt that this was a good experience. Everything worked very smoothly (besides the occasional technology hiccup of course). I felt that the training I received was equal to or even better than previous in-person precepting experiences.” –Pharmacist HPT

“[Virtual supervision was] great, easy to review data and discuss with attending over Microsoft Teams.” – Physician HPT

“Far as technology, I was satisfied. I was able to easily access my supervisors this way and meet with supervisors at different clinics, teleworking, and with busy schedules.”

– Psychologist HPT

“Helpful preceptor. Experienced a day when the phones were down.” – Nurse HPT

Representative Comments of Virtual Supervision Influenced by the Clinical Supervisor:

“It was wonderful to have my supervisors so accessible. Virtual supervision allowed me to effectively engage in a major rotation during half of a day and engage in my minor rotation the other half of the day. Based on the way patient care fell (i.e., group schedules, appointment times), it would have been difficult to attend all of these as they are across the medical center campus. Virtual supervision truly streamlined my internship. – Psychologist HPT

“Value of virtual supervision varied by supervisor and often matched their in-person supervision style; if they were conscientious in person, that remained in remote format, if not, that was also true for both formats.” – Psychologist HPT

“It was a nice change of pace my preceptor was amazing and so kind and welcoming.” – Nurse HPT

“Dr. R was ABSOLUTELY amazing. She was very resourceful. I participated in Topic Discussions, MH travel team, VVCs [VA video connect], F2F appointments, Team Huddles, AAPP webinars and learn much more than I could ever imagine. No setbacks and she was always available. Really in a class of her own. – Physician HPT

“Somewhat challenging due to all duties demanded of preceptor - she was willing but super busy.” – Physician HPT

Depending on the type of training experience, the amount of time spent in virtual supervision varied among HPTs. Frequency ranged from occasional sessions in special circumstances to weekly sessions where almost all supervision was conducted virtually. For physician HPTs, virtual supervision most occurred within specific rotations utilizing telehealth clinics, such as dermatology, psychiatry, and stroke care. Pharmacy and nursing HPTs experienced virtual supervision during specific clinic rotations such as anticoagulation and ambulatory care, whereas psychology and social work HPTs received virtual supervision during cognitive behavioral therapy training experiences.

#### Representative comments of virtual supervision influenced by amount of time spent in virtual supervision

“A few weeks into my internship/training I had to do [virtual] supervision because I had tested positive for Covid-19. I called in with my supervisor throughout the week and she had given me assigned tasks. My supervisor was very understanding and gave me stuff to do that was beneficial and not just busy work.” – Associated Health HPT

“Facetimed my supervisor during patient home visit. Experience was overall good and allowed me to practice independently, but still with supervision/backup as necessary.” – Physician HPT

“I worked as part of a virtual BHIP [Behavioral Health Interdisciplinary Program] team during my general mental health clinic rotation. As part of that experience, I received regular supervision from my mentor/preceptor.” – Nurse HPT

## Discussion

This study documents HPTs’ involvement with telehealth and virtual supervision in VA, the largest interprofessional clinical training environment in the U.S. The large sample of HPTs in this analysis was stratified by profession to examine varied responses influenced by professional backgrounds and diverse training models. The distribution of HPT professions in the sample was not significantly different from the percent of VA-funded HPTs nationally. Respondents represented 133 VA facilities reflecting the geographic diversity of the VA system.

In the overall sample, approximately one-third reported participating in telehealth and one-fourth in virtual supervision, with statistically significant differences by profession. The literature documents differences in telehealth use and virtual supervision by profession, and furthermore between specialties within a profession [27–29]. Primary care physicians and nurse practitioners may use telehealth to manage chronic disease and provide follow-up [27, 28], whereas psychiatrists may use telehealth for consultation and diagnosis [27–29]. Psychologists may use telehealth to provide online therapy [30], and physical therapists may use telehealth to teach and monitor rehabilitation exercise [31]. Other factors influencing telehealth use and cited in the literature include reimbursement issues, lack of training, technology issues, and concerns about privacy and quality of care. Reimbursement issues do not apply in the VA training environment but HPTs in our study did identify technology and supervisor traits as prevalent themes shaping perceptions of these practices within the context of the training environment. A notable theme in our study is that soft personality skills e.g. kindness, availability, supportiveness are important supervisory traits for virtual supervision [1, 32–34]. Musharyanti (2024) found that these soft skills and the amount of attention, recognition, appreciation and flexibility provided by clinical supervisors are important to the success of clinical education for nursing students [35]. Future work could address personality traits and behaviors shaping the supervisor-supervisee relationship across health care professions and their impact on the effectiveness of teaching in the virtual environment.

The pandemic drastically shifted healthcare delivery towards virtual visits. A national study of 36 million working-age individuals with private insurance claims data showed that the percentage of telemedicine encounters increased from 0.3% of all encounters (pre-pandemic) to 23.6% of all encounters (first year of the pandemic) resulting in an overall increased utilization of telemedicine by 766% [36]. Post-pandemic, telehealth usage remains steady [37]. As healthcare pivots towards virtual visits, our findings of positive perceptions by HPTs and supervisors promote its enduring relevance in the VA healthcare environment.

The strengths of this study include its large sample size, the inclusion of multiple HPT professions and the diversity of training experiences, which together provide a more comprehensive view of HPT involvement in telehealth and virtual supervision for the largest clinical training environment in the U.S. Study weaknesses include the low estimated overall response rate on the survey and the limited generalizability to all HPTs. The TSS was designed to measure satisfaction across multiple trainee experiences, but not all virtual supervision responses correlated with the TSS open-ended question, requiring unclear responses to be screened out of the sample. Soliciting more targeted feedback through a comprehensive instrument could enhance understanding of how to best structure telehealth and virtual supervision practices for HPTs to maximize educational quality and patient outcomes. A comprehensive instrument could also delve deeper into the type of telemedicine visit (audio vs video), how they are used by different HPT professions, and their impacts on virtual supervision practices.

To ensure the highest quality of care and education, further study is required related to the best balance of telehealth, virtual supervision and traditional models [9, 38]. Future research should consider use of a comprehensive instrument measuring multiple dimensions of telehealth and virtual supervision to identify influencing factors.

## Conclusion

The growing use of telehealth and the changing landscape of care delivery are driving transformation of HPT supervision. More work is needed to define clinical educational and outcomes for HPTs utilizing telehealth and virtual supervision. The VA’s interprofessional clinical learning environment, in partnership with academic institutions, provides a fertile foundation for exploring and evaluating new enhanced models of telehealth and virtual supervision.

## Data Availability

The dataset used in this evaluation are maintained by the United States Department of Veterans Affairs, Office of Academic Affiliations. These data are not publicly available but may be requested through the Freedom of Information Act.

## Abbreviations

AY: Academic year
FY: Fiscal year
HPE: Health professions education
HPT: Health profession trainee
LPN/LVN/NA: Licensed practical nurses/licensed vocational nurses, nurse assistants
OAA: Office of Academic Affiliations
TSS: Trainee Satisfaction Survey
U.S.: United States
VA: Department of Veterans Affairs
VHA: Veterans Health Administration
VVC: VA video connect

## Footnotes

1 HPTs may be assigned to the VA for clinical training across AYs due to administrative scheduling for clinical training rotations by academic institutions (universities/colleges).

## References

[1] Martin P, Lizarondo L, Kumar S. A systematic review of the factors that influence the quality and effectiveness of telesupervision for health professionals. J Telemed Telecare. 2018;24(4):271–81. 10.1177/1357633X17698868. 28387603 10.1177/1357633X17698868

[2] Martin P, Kumar S, Lizarondo L. Effective use of technology in clinical supervision. Internet Interv. 2017;8:35–9. 10.1016/j.invent.2017.03.001. 30135826 10.1016/j.invent.2017.03.001 PMC6096199

[3] Brandoff R, Lombardi R. Miles apart: two art therapists’ experience of distance supervision. Art Ther. 2012;29(2):93–6.

[4] Perle JG, Zheng W. A Primer for Understanding and Utilizing Telesupervision with Healthcare Trainees. J Technol Behav Sci. Published online May 19, 2023:1–7. 10.1007/s41347-023-00322-5. 10.1007/s41347-023-00322-5 PMC10196304 37362064

[5] Lawrence K, Nov O, Mann D, Mandal S, Iturrate E, Wiesenfeld B. The impact of telemedicine on physicians’ after-hours electronic health record “Work Outside Work” During the COVID-19 Pandemic: Retrospective Cohort Study. JMIR Med Inform. 2022;10(7):e34826. 10.2196/34826. 35749661 10.2196/34826 PMC9337620

[6] Northcraft H, Bai J, Griffin AR, Hovsepian S, Dobalian A. Association of the COVID-19 Pandemic on VA Resident and Fellow Training Satisfaction and Future VA Employment: A Mixed Methods Study. J Grad Med Educ. 2022;14(5):593–8. 10.4300/JGME-D-22-00168.1. 36274776 10.4300/JGME-D-22-00168.1 PMC9580318

[7] Balut MD, Wyte-Lake T, Steers WN, et al. Expansion of telemedicine during COVID-19 at a VA specialty clinic. Healthc (Amst). 2022;10(1):100599. 10.1016/j.hjdsi.2021.100599. 34999492 10.1016/j.hjdsi.2021.100599 PMC8616735

[8] Doraiswamy S, Abraham A, Mamtani R, Cheema S. Use of telehealth during the COVID-19 pandemic: scoping review. J Med Internet Res. 2020;22(12):e24087. 10.2196/24087. 33147166 10.2196/24087 PMC7710390

[9] Andino JJ, Eyrich NW, Boxer RJ. Overview of telehealth in the United States since the COVID-19 public health emergency: a narrative review. Mhealth. 2023;9:26. 10.21037/mhealth-23-15. 37492124 10.21037/mhealth-23-15 PMC10364039

[10] United States Department of Veterans Affairs. >VHA Telehealth Services. https://connectedcare.va.gov/sites/default/files/OT_va-telehealth-factsheet-2019-01.pdf. Accessed 17 Jul 2024.

[11] Heyworth L, Shah N, Galpin K. 20 years of telehealth in the veterans health administration: taking stock of our past and charting our future. J Gen Intern Med. 2024;39(Suppl 1):5–8. 10.1007/s11606-024-08617-w. 10.1007/s11606-024-08617-w PMC10937874 38378981

[12] United States Department of Veterans Affairs. VA Telehealth. https://telehealth.va.gov/type/clinic. Accessed 17 Jul 2024.

[13] Lutes T. With telehealth, Veterans can access care when and where they need it. VA News. https://news.va.gov/108453/telehealth-access-care-when-where-need/. September 19, 2022. Accessed 17 Jul 2024.

[14] Office of Academic Affiliations. Health Professions Education Statistics, Academic Year 2022–2023. U.S. Department of Veterans Affairs., https://www.va.gov/OAA/docs/OAA_Stats_Sheet_AY_2022-2023.pdf. Accessed 22 Jan 2025.

[15] Office of Academic Affiliations. VA Memorandum to Associate Chiefs of Staff for Education/Designated Education Officers, Update on Virtual Supervision. Published online September 8, 2022.

[16] Office of Academic Affiliations. VHA Directive 1400.01 Supervision of Physician, Dental, Optometry, Chiropractic, and Podiatry Residents; 2019. https://www.va.gov/vhapublications/viewpublication.asp?pub_id=8579. Accessed 22 Jan 2025.

[17] SurveyMonkey Inc. San Mateo, California, USA. http://www.surveymonkey.com.

[18] Griffin AR, Bai J, Northcraft H, Dobalian A. Veterans affairs training experiences during the COVID-19 pandemic-trainee satisfaction and interest in future veterans affairs employment: a national survey. SAGE Open Nurs. 2024;10:23779608241261616. 10.1177/23779608241261617. 39185508 10.1177/23779608241261617 PMC11342328

[19] Northcraft H, Bai J, Griffin AR, Dobalian A. Dental and dental hygienist trainee satisfaction with their veterans affairs clinical training experiences during the COVID-19 pandemic. BMC Med Educ. 2024;24(1):655. 10.1186/s12909-024-05628-3. 38862948 10.1186/s12909-024-05628-3 PMC11167822

[20] Henrikson NB, Moldestad M, Maynard C, et al. Inclination to pursue Veterans Health Administration for primary care practice: survey of medical residents. Front Health Serv. 2024;4:1394072. 10.3389/frhs.2024.1394072. 39091517 10.3389/frhs.2024.1394072 PMC11291321

[21] Department of Veterans Affairs O of R& D. Program Guide: 1200.21 VHA Operations Activities That May Constitute Research.; 2019. https://www.research.va.gov/resources/policies/ProgramGuide-1200-21-VHA-Operations-Activities.pdf. Accessed 29 Aug 2024.

[22] IBM Corporation. SPSS Statistics for Windows, Version 29.0.1. https://www.google.com/url?sa=t&rct=j&q=&esrc=s&source=web&cd=&cad=rja&uact=8&ved=2ahUKEwiD0-,T2rYqLAxV1GjQIHTHcH_QQFnoECBMQAQ&url=https%3A%2F%2Fwww.ibm.com%2Fsupport%2Fpages%2Fdownloading-ibm-spss-statistics-2901&usg=AOvVaw27ZYmpDhsp7ewc_-XnWfJZ&opi=89978449. Accessed 22 Jan 2025.

[23] Palinkas LA, Mendon SJ, Hamilton AB. Innovations in Mixed Methods Evaluations. Annu Rev Public Health. 2019;40:423–42. 10.1146/annurev-publhealth-040218-044215. 30633710 10.1146/annurev-publhealth-040218-044215 PMC6501787

[24] St George SM, Harkness AR, Rodriguez-Diaz CE, Weinstein ER, Pavia V, Hamilton AB. Applying Rapid Qualitative Analysis for Health Equity: Lessons Learned Using “EARS” With Latino Communities. Int J Qual Methods. 2023;22. 10.1177/16094069231164938. 10.1177/16094069231164938 PMC10923582 38463016

[25] Taylor B, Henshall C, Kenyon S, Litchfield I, Greenfield S. Can rapid approaches to qualitative analysis deliver timely, valid findings to clinical leaders? A mixed methods study comparing rapid and thematic analysis. BMJ Open. 2018;8(10):e019993. 10.1136/bmjopen-2017-019993. 10.1136/bmjopen-2017-019993 PMC6194404 30297341

[26] Bingham AJ. From Data Management to Actionable Findings: A Five-Phase Process of Qualitative Data Analysis. Int J Qual Methods. 2023;22. 10.1177/16094069231183620.

[27] Bauer JE, White D. Telehealth nursing: the impact of technology on healthcare delivery. J Telemed Telecare. 2020;26(8):452–61.30975047

[28] Bashshur RL, Shannon GW, Krupinski EA. The role of telemedicine in the future of health care delivery. Telemed e-Health. 2013;19(1):1–10.

[29] Kane CK, Gillis K. The use of Telemedicine by Physicians: Still the Exception Rather Than the Rule. Health Aff. 2018;37(12):1923–30. 10.1377/hltaff.2018.0507. 10.1377/hlthaff.2018.05077 30633670

[30] Torous J, et al. The COVID-19 pandemic and the use of telehealth for mental health: A rapid review. J Psychiatr Res. 2020;131:234–6.

[31] Tse J, Lee P. Telemedicine for rehabilitation: an overview of telehealth in allied health professions. J Rehabil Res Dev. 2018;55(1):39–46.

[32] Lafleur A, Gagné M, Paquin V, Michaud-Couture C. How to Convince Clinicians that “Soft” Skills Save Lives? Practical Tips to Use Clinical Studies to Teach Physicians’ Roles. MedEdPublish. 2016;2019(8):119. 10.15694/mep.2019.000119.1. 10.15694/mep.2019.000119.1 PMC10712525 38089257

[33] Cameron M, Ray R, Sabesan S. Remote supervision of medical training via videoconference in northern Australia: a qualitative study of the perspectives of supervisors and trainees. BMJ Open. 2015;5(3):e006444. 10.1136/bmjopen-2014-006444. 10.1136/bmjopen-2014-006444 PMC4368981 25795687

[34] Rothwell C, Kehoe A, Farook SF, Illing J. Enablers and barriers to effective clinical supervision in the workplace: a rapid evidence review. BMJ Open. 2021;11(9):e052929. 10.1136/bmjopen-2021-052929. 10.1136/bmjopen-2021-052929 PMC8479981 34588261

[35] Musharyanti, L. The Role of Clinical Supervisors in Clinical Nursing Education: A Literature Review. Jurnal Aisyah: Jurnal Ilmu Kesehatan. 2024;9(2). 10.30604/jika.v912.2485.

[36] Shaver J. The State of Telehealth Before and After the COVID-19 Pandemic. Prim Care. 2022;49(4):517–30. 10.1016/j.pop.2022.04.002. 36357058 10.1016/j.pop.2022.04.002 PMC9035352

[37] Assistant Secretary for Planning and Evaluation Office of Health Policy. Updated National Survey Trends in Telehealth Utilization and Modality (2021–2022); 2023. https://aspe.hhs.gov/sites/default/files/documents/7d6b4989431f4c70144f209622975116/household-pulse-survey-telehealth-covid-ib.pdf. Accessed 15 Jul 2024. 38913813

[38] Bellini MI, Pengel L, Potena L, Segantini L, ESOT COVID-19 Working Group. COVID-19 and education: restructuring after the pandemic. Transpl Int. 2021;34(2):220–223. 10.1111/tri.13788. 10.1111/tri.13788 PMC7753626 33205410

